# Burnout Syndrome Among Pediatric Residents at a Children’s Hospital in Madrid

**DOI:** 10.1192/j.eurpsy.2025.1594

**Published:** 2025-08-26

**Authors:** S. Jaramillo, B. Martínez-Núñez, M. Faya-Barrios, M. Graell

**Affiliations:** 1 Department of Psychiatry, Hospital Infantil Universitario Niño Jesús, Madrid, Spain

## Abstract

**Introduction:**

Burnout Syndrome (BOS) is a state of exhaustion due to chronic workplace stress, characterized by emotional exhaustion (EE), depersonalization (DP), and a diminished sense of personal accomplishment (PA). BOS is significant among healthcare professionals worldwide, and a critical issue in medical residency, raising concerns about mental health, patient care and safety.

**Objectives:**

This study aimed to evaluate the prevalence and severity of BOS among pediatric medical residents at a children’s hospital in Madrid, Spain. The association between demographic and occupational factors, such as gender, residency year, and night shifts per month, and the presence of BOS was also analyzed.

**Methods:**

An observational, cross-sectional study was conducted in September 2024, using a survey distributed to pediatric residents. The survey included demographic and occupational data and the Maslach Burnout Inventory Human Services Survey (MBI-HSS), which assesses burnout through EE, DP and PA. Perception of institutional burnout prevention and support programs was also evaluated.

**Results:**

The response rate was 81.8% (45/55), the majority of respondents were female (77.8%), aged between 24-32 years (mean 26.8), from all levels of training. Burnout scores were abnormal in 55.6%, and 11.1% met criteria for high levels of burnout. Emotional exhaustion was the most affected dimension, over half (57.8%) scoring in the high-degree for EE, followed by 40% for high DP and 28.9% for low PA. No significant associations were found between gender, residency year, or night shifts and burnout levels. However, 66.7% of the respondents perceived insufficient institutional burnout prevention programs or support.

**Conclusions:**

The study confirmed a high prevalence of BOS among pediatric residents. These results emphasize the urgent need for targeted interventions to prevent and address burnout and improve health-care professionals well-being. Further research is needed to explore factors contributing to burnout and effective strategies for mitigating burnout among medical residents.

**Disclosure of Interest:**

None Declared

